# Supplementary material for: Double-helix singularity and vortex–antivortex annihilation in space-time helical pulses
Source: Nanophotonics. 2024 Oct 31;14(6):741–7. doi: 10.1515/nanoph-2024-0480 (PMC11964190; doi:10.1515/nanoph-2024-0480)
Supplement: Supplementary file 1 — Supplementary Material Details [file j_nanoph-2024-0480_suppl_001.pdf]

Supplementary Information for

Double-Helix Singularity and Vortex-Antivortex annihilation in Space-Time Helical Pulses

Shuai Shi[1], Ren Wang[1,2*], Minhui Xiong[1], Qinyu Zhou[1], Bing-Zhong Wang[1] and Yijie Shen[3,4*]

[1] Institute of Applied Physics, University of Electronic Science and Technology of China, Chengdu 611731, China

[2] Yangtze Delta Region Institute (Huzhou), University of Electronic Science and Technology of China, Huzhou 313098, China

[3] Centre for Disruptive Photonic Technologies, School of Physical and Mathematical Sciences, Nanyang Technological University, Singapore 637378, Singapore

[4] School of Electrical and Electronic Engineering, Nanyang Technological University, Singapore 637378, Singapore

*Correspondence to: Ren Wang (rwang@uestc.edu.cn) and Yijie Shen (yijie.shen@ntu.edu.sg)

Supplementary Note 1:
Derivation of TE and TM helical pulses.

To obtain the electric and magnetic fields for the helical pulses, we use Hertz's method. First, we find an appropriate scalar-generating function $f(\mathbf{r},t)$ that satisfies Helmholtz's wave equation in vacuum

$$\left(\nabla^2 - \frac{1}{c^2}\frac{\partial^2}{\partial t^2}\right) f(\mathbf{r},t) = 0 \qquad \text{(S1)}$$

Where $\mathbf{r} = (r,\theta,z)$ are cylindrical coordinates, t is time, $c = 1/\sqrt{\mu_0\varepsilon_0}$ is the speed of light, and the ε_0 and μ_0 are the permittivity and permeability of the medium. Following Ziolkowski, localized finite-energy pulses can be obtained as superpositions of "electromagnetic directed-energy pulse trains", we start from the scalar generating function in EDEPT [1] method. Hillion [2] has shown that the wave equation is solved by the set of functions

$$f(\mathbf{r},t) = \frac{f(s)}{b + \mathrm{i}(z \mp ct)}, s = \frac{\rho^2}{b + \mathrm{i}(z \mp ct)} - \mathrm{i}(z \pm ct) \qquad \text{(S2)}$$

When $f(s)$ is set equal to $ab\varphi_0/(s+a)$, we obtain the Ziolkowski wavefunction[1]

$$f_z = \frac{ab\varphi_0}{\rho^2 + \left[a - i(z \pm ct)\right]\left[b + i(z \mp ct)\right]} \qquad \text{(S3)}$$

We now wish to generate solutions of the wave equation with azimuthal dependence. We note that

$$\frac{g}{b + i(z \mp ct)} f_z, g = x, y, x \pm iy \qquad \text{(S4)}$$

are solutions of the wave equation, as are

$$\frac{h}{\left[b + i(z \mp ct)\right]^2} f_z, h = xy, x^2 - y^2, (x \pm iy)^2 \qquad \text{(S5)}$$

The wavefunctions obtained by replacing f_z by $f(s)/b + \mathrm{i}(z \mp ct)$ in Eq.(S4) and Eq.(S5) are also solutions of the wave equation. We are particularly interested in azimuthal dependence of the form $\mathrm{e}^{il\theta}$, where l is a positive or negative integer. We note that $x + iy = \rho e^{il\theta}$, and can verify by differentiation that [3]

$$\left[\frac{\rho}{b + \mathrm{i}(z - ct)}\right]^{|l|} \mathrm{e}^{il\theta} \frac{f(s)}{b + \mathrm{i}(z - ct)}, s = \frac{\rho^2}{b + \mathrm{i}(z - ct)} - \mathrm{i}(z + ct) \qquad \text{(S6)}$$

is the solution of the wave equation for arbitrary l and any twice-differentiable function $f(s)$. In quantum mechanics the factor $\mathrm{e}^{il\theta}$ would be associated with angular momentum $l\hbar$ about the polar (z) axis. Then, the exact solution of $f(\mathbf{r},t)$ can be given by the modified power spectrum method[4], when $f(s)$ is set equal to $f_0/(s+a)$, a, b is set equal to q_2, q_1, respectively, we can obtain the generating wavefunction

$$f = \left(\frac{\rho}{q_1 + \mathrm{i}\tau}\right)^{|l|} \mathrm{e}^{il\theta} \frac{f_0}{\rho^2 + (q_1 + \mathrm{i}\tau)(q_2 - \mathrm{i}\sigma)} \tag{S7}$$

Where $\tau = z - ct$, $\sigma = z + ct$, and f_0 is a normalized constant. When compared to a Gaussian beam, the parameters q_1 and q_2 represent respectively the effective wavelength and the "Rayleigh range" or depth of the focal region. In particular, the value of the ratio q_2 / q_1 indicates whether the pulse is collimated ($q_2 / q_1 \gg 1$) or strongly focused.

Next, we construct the Hertz potential, solutions to Maxwell's equations follow naturally from the scalar wave equation solutions. For fulfilling the toroidal symmetric and azimuthally polarized structure, defining the electric, $\Pi_e = f\hat{\mathbf{n}}$, or magnetic, $\Pi_h = f\hat{\mathbf{n}}$. Hertz potential along the arbitrary direction $\hat{\mathbf{n}}$, one readily obtains fields that are TE or TM with respect to $\hat{\mathbf{n}}$. For instance, if $\hat{\mathbf{n}} = \hat{\mathbf{z}}$, then the TE polarization

$$\begin{cases} E(\mathbf{r},t) = -Z_0 \nabla \times \partial_{ct} \mathbf{\Pi}_h = -\hat{\boldsymbol{\rho}} \frac{1}{\rho} \sqrt{\frac{\mu_0}{\varepsilon_0}} \partial_\theta \partial_{ct} f(\mathbf{r},t) + \hat{\boldsymbol{\theta}} \sqrt{\frac{\mu_0}{\varepsilon_0}} \partial_\rho \partial_{ct} f(\mathbf{r},t) \\ H(\mathbf{r},t) = \nabla(\nabla \cdot \mathbf{\Pi}_h) - \partial_{ct}^2 \mathbf{\Pi}_h = \hat{\boldsymbol{\rho}} \partial_\rho \partial_z f(\mathbf{r},t) + \hat{\boldsymbol{\theta}} \frac{1}{\rho} \partial_\theta \partial_z f(\mathbf{r},t) + \hat{\mathbf{z}} \left(\partial_z^2 - \partial_{ct}^2\right) f(\mathbf{r},t) \end{cases} \tag{S8}$$

Then, in a cylindrical coordinate system, the exact solutions of transverse electric (TE) field components are given by the expressions

$$E_\rho = \frac{f_0 l e^{il\theta}}{\rho} \sqrt{\frac{\mu_0}{\varepsilon_0}} \left(\frac{\rho}{q_1 + i\tau}\right)^{|l|} \left\{ \frac{(q_2 + q_1 - 2ict)}{\left[\rho^2 + (q_1 + i\tau)(q_2 - i\sigma)\right]^2} + \frac{|l|}{\left[\rho^2 + (q_1 + i\tau)(q_2 - i\sigma)\right](q_1 + i\tau)} \right\}$$

(S9)

$$\begin{aligned} E_\theta = i f_0 \sqrt{\frac{\mu_0}{\varepsilon_0}} e^{il\theta} \left(\frac{\rho}{q_1 + i\tau}\right)^{|l|} \Bigg\{ & \frac{4\rho(q_2 + q_1 - 2ict)}{\left[\rho^2 + (q_1 + i\tau)(q_2 - i\sigma)\right]^3} + \frac{|l|(q_2 + q_1 - 2ict)}{\rho\left[\rho^2 + (q_1 + i\tau)(q_2 - i\sigma)\right]^2} \\ & - \frac{2|l|\rho}{\left[\rho^2 + (q_1 + i\tau)(q_2 - i\sigma)\right]^2 (q_1 + i\tau)} + \frac{l^2}{\left[\rho^2 + (q_1 + i\tau)(q_2 - i\sigma)\right]\rho(q_1 + i\tau)} \Bigg\} \end{aligned} \tag{S10}$$

$$\begin{aligned} H_\rho = f_0 e^{il\theta} \left(\frac{\rho}{q_1 + i\tau}\right)^{|l|} \Bigg\{ & \frac{-il^2}{\left[\rho^2 + (q_1 + i\tau)(q_2 - i\sigma)\right]\rho(q_1 + i\tau)} + \frac{4\rho\left[(q_2 - q_1)i + 2z\right]}{\left[\rho^2 + (q_1 + i\tau)(q_2 - i\sigma)\right]^3} \\ & - \frac{\left[(q_2 - q_1)i + 2z\right]|l|}{\rho\left[\rho^2 + (q_1 + i\tau)(q_2 - i\sigma)\right]^2} + \frac{2i\rho|l|}{\left[\rho^2 + (q_1 + i\tau)(q_2 - i\sigma)\right]^2 (q_1 + i\tau)} \Bigg\} \end{aligned} \tag{S11}$$

$$H_\theta = \left(\frac{\rho}{q_1 + i\tau}\right)^{|l|} l f_0 e^{il\theta} \left\{ -\frac{i\left[(q_2 - q_1)i + 2z\right]}{\left[\rho^2 + (q_1 + i\tau)(q_2 - i\sigma)\right]^2} + \frac{|l|}{\left[\rho^2 + (q_1 + i\tau)(q_2 - i\sigma)\right]\rho(q_1 + i\tau)} \right\}$$

(S12)

$$H_z = 2f_0 e^{il\theta}\left(\frac{\rho}{q_1+i\tau}\right)^{|l|}\left\{-\frac{2}{\left[\rho^2+(q_1+i\tau)(q_2-i\sigma)\right]^2}+\frac{(q_2+q_1-2ict)^2+\left[(q_2-q_1)i+2z\right]^2}{\left[\rho^2+(q_1+i\tau)(q_2-i\sigma)\right]^3}\right.$$
$$\left.+\frac{|l|\left[(q_2+q_1-2ict)+i\left[(q_2-q_1)i+2z\right]\right]}{\left[\rho^2+(q_1+i\tau)(q_2-i\sigma)\right]^2(q_1+i\tau)}\right\}$$

(S13)

For this pulse solution, the electric field is azimuthally and radially polarized, and the magnetic field is along the radial, azimuthal and longitudinal directions.

In particular, TM polarization

$$\begin{cases} E(\mathbf{r},t)=\nabla(\nabla\cdot\mathbf{\Pi_e})-\partial_{ct}^2\mathbf{\Pi_e}=\hat{\boldsymbol{\rho}}\partial_\rho\partial_z f(\mathbf{r},t)+\hat{\boldsymbol{\theta}}\frac{1}{\rho}\partial_\theta\partial_z f(\mathbf{r},t)+\hat{\mathbf{z}}\left(\partial_z^2-\partial_{ct}^2\right)f(\mathbf{r},t) \\ H(\mathbf{r},t)=Y_0\nabla\times\partial_{ct}\mathbf{\Pi_e}=\hat{\boldsymbol{\rho}}\frac{1}{\rho}\sqrt{\frac{\varepsilon_0}{\mu_0}}\partial_\theta\partial_{ct}f(\mathbf{r},t)-\hat{\boldsymbol{\theta}}\sqrt{\frac{\varepsilon_0}{\mu_0}}\partial_\rho\partial_{ct}f(\mathbf{r},t)\end{cases} \tag{S14}$$

Then in a cylindrical coordinate system, the exact solutions of transverse magnetic (TM) field components are given by the expressions

$$E_\rho = f_0 e^{il\theta}\left(\frac{\rho}{q_1+i\tau}\right)^{|l|}\left\{\frac{-il^2}{\left[\rho^2+(q_1+i\tau)(q_2-i\sigma)\right]\rho(q_1+i\tau)}+\frac{4\rho\left[(q_2-q_1)i+2z\right]}{\left[\rho^2+(q_1+i\tau)(q_2-i\sigma)\right]^3}\right.$$
$$\left.-\frac{\left[(q_2-q_1)i+2z\right]|l|}{\rho\left[\rho^2+(q_1+i\tau)(q_2-i\sigma)\right]^2}+\frac{2i\rho|l|}{\left[\rho^2+(q_1+i\tau)(q_2-i\sigma)\right]^2(q_1+i\tau)}\right\} \tag{S15}$$

$$E_\theta = \left(\frac{\rho}{q_1+i\tau}\right)^{|l|} l f_0 e^{il\theta}\left\{-\frac{i\left[(q_2-q_1)i+2z\right]}{\left[\rho^2+(q_1+i\tau)(q_2-i\sigma)\right]^2}+\frac{|l|}{\left[\rho^2+(q_1+i\tau)(q_2-i\sigma)\right]\rho(q_1+i\tau)}\right\}$$

(S16)

$$E_z = 2f_0 e^{il\theta}\left(\frac{\rho}{q_1+i\tau}\right)^{|l|}\left\{-\frac{2}{\left[\rho^2+(q_1+i\tau)(q_2-i\sigma)\right]^2}+\frac{(q_2+q_1-2ict)^2+\left[(q_2-q_1)i+2z\right]^2}{\left[\rho^2+(q_1+i\tau)(q_2-i\sigma)\right]^3}\right.$$
$$\left.+\frac{|l|\left[(q_2+q_1-2ict)+i\left[(q_2-q_1)i+2z\right]\right]}{\left[\rho^2+(q_1+i\tau)(q_2-i\sigma)\right]^2(q_1+i\tau)}\right\}$$

(S17)

$$H_\rho = \frac{-f_0 l e^{il\theta}}{\rho}\sqrt{\frac{\varepsilon_0}{\mu_0}}\left(\frac{\rho}{q_1+i\tau}\right)^{|l|}\left\{\frac{(q_2+q_1-2ict)}{\left[\rho^2+(q_1+i\tau)(q_2-i\sigma)\right]^2}+\frac{|l|}{\left[\rho^2+(q_1+i\tau)(q_2-i\sigma)\right](q_1+i\tau)}\right\}$$

(S18)

$$H_\theta = -if_0\sqrt{\frac{\varepsilon_0}{\mu_0}}e^{il\theta}\left(\frac{\rho}{q_1+i\tau}\right)^{|l|}\left\{\frac{4\rho(q_2+q_1-2ict)}{\left[\rho^2+(q_1+i\tau)(q_2-i\sigma)\right]^3}+\frac{|l|(q_2+q_1-2ict)}{\rho\left[\rho^2+(q_1+i\tau)(q_2-i\sigma)\right]^2}\right.$$
$$\left.+\frac{l^2}{\left[\rho^2+(q_1+i\tau)(q_2-i\sigma)\right]\rho(q_1+i\tau)}-\frac{2|l|\rho}{\left[\rho^2+(q_1+i\tau)(q_2-i\sigma)\right]^2(q_1+i\tau)}\right\} \tag{S19}$$

Meanwhile, we can also obtain TM solutions directly by the simple transformation relationship between TE and TM solutions by

$$E_{TM} = \sqrt{\frac{\mu_0}{\varepsilon_0}} H_{TE} \quad H_{TM} = -\sqrt{\frac{\varepsilon_0}{\mu_0}} E_{TE} \tag{S20}$$

For this pulse solution, exactly the opposite of TE, the magnetic field is azimuthally and radially polarized, and the electric field is along the radial, azimuthal and longitudinal directions.

The rotational relationship between the real and imaginary parts can be demonstrated through straightforward formulas. Let us take the electric field of the TE mode as an example. In polar coordinates, Eq.(S15) and Eq.(S16) allow for the separation of the azimuthal angle variable. At this point, Eq.(S15) and Eq.(S16) can be expressed in the following form

$$h(\theta,\rho,z,t) = e^{il\theta} g(\rho,z,t) \tag{S21}$$

The real and imaginary parts can be written as

$$\begin{aligned} h_{\mathrm{re}}(\theta,\rho,z,t) &= \cos(l\theta) g_{\mathrm{re}}(\rho,z,t) - \sin(l\theta) g_{\mathrm{im}}(\rho,z,t) \\ h_{\mathrm{im}}(\theta,\rho,z,t) &= \sin(l\theta) g_{\mathrm{re}}(\rho,z,t) + \cos(l\theta) g_{\mathrm{im}}(\rho,z,t) \end{aligned} \tag{S22}$$

Based on the properties of trigonometric functions, we can readily obtain

$$\begin{aligned} h_{\mathrm{im}}\left(\theta+\frac{\pi}{2l},\rho,z,t\right) &= \sin\left(l\theta+\frac{\pi}{2}\right) g_{\mathrm{re}}(\rho,z,t) + \cos\left(l\theta+\frac{\pi}{2}\right) g_{\mathrm{im}}(\rho,z,t) \\ &= \cos(l\theta) g_{\mathrm{re}}(\rho,z,t) - \sin(l\theta) g_{\mathrm{im}}(\rho,z,t) \\ &= h_{\mathrm{re}}(\theta,\rho,z,t) \end{aligned} \tag{S23}$$

In the context of vector transformations in cylindrical coordinates, when the azimuthal angle undergoes a change, the vector bases simultaneously undergo rotation. Therefore, the rotational relationship between the real and imaginary parts signifies that both the vector direction and the field structure undergo rotation concurrently.

Now let's prove the necessity of $l \le 2$, which requires only a few simple steps. Take Eq.(S9) as an example. When we set ρ to infinity ($\rho \to \infty$), Eq.(S9) can be transformed into a different form

$$E_\rho \sim \rho^{3-|l|} \tag{S24}$$

It is evident that when $l = 3$, E_ρ approaches a constant as ρ goes to infinity. In this case, due to the infinite integration space, the calculated energy result is positive infinity. When $l > 3$, the E_ρ component increases infinitely with the increasing radius, and the calculated energy result is also infinite, which does not meet the limiting condition of finite energy. Therefore, it is necessary that $l \le 2$.

Supplementary Note 2:
Propagation characteristics of TE helical pulses.

Two different pulses can be constructed respectively from the real and imaginary parts of the complex electromagnetic fields of Eq.(S9)- Eq.(S13), both types of which are exact solutions to Maxwell's equations. In fact, through some simple deductions, it can be easily found that the imaginary part field can be obtained by rotating the real part field by $\pi / 2l$. So when $l = 1$, the imaginary part field is the real part field rotating by $\pi / 2$. For focused FD (Flying doughnuts) Pulses, $l = 0$, situation is different, it is rotationally symmetric. The real/imaginary part is single/1½ cycle in the electric field and 1½/ single cycle in the magnetic field at the focus ($t = 0$). Thus, the real part is referred to as the single-cycle pulse, and the imaginary one is referred to as the 1½-cycle pulse. However, significant temporal reshaping of the pulses occurs as they propagate along *z*, the single-cycle (1½-cycle) pulse transforms to the 1½-cycle (single-cycle) pulse due to the Gouy phase shift. In order to illustrate the transformation more clearly, the field of the helical pulses at different moments is calculated theoretically according to equations, and the electric field value on the cross-section is extracted. Several locations in space are selected to record the time domain waveforms of the helical pulses propagating in free space. Here, the time domain waveforms are recorded and normalized with their maximum value of their range. It could show the spatial-temporal transformations that both the real and imaginary pulses of the TE helical pulses with different order undergo as they propagate along *z*. For the case $l = 1$, the imaginary pulse on the $x = 0$ plane and the real pulse on the $y = 0$ plane are the same.

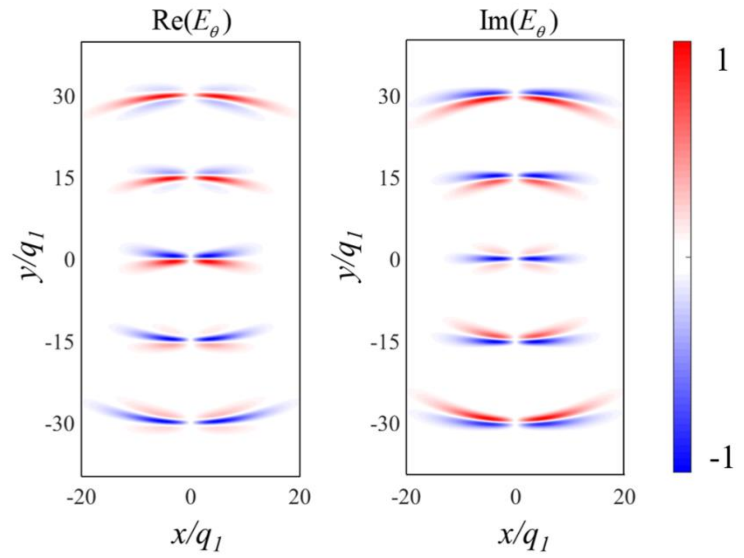

Figure S1 Time domain waveforms and the normalized electric field components on the *y*-*z* cross-section of the real and imaginary parts of the TE $l = 0$ helical pulses. As they propagate *z*-direction, a transition between single-cycle and 1½-cycle pulses is demonstrated. Due to the Gouy phase shift, the real pulse (1½-cycle) shifts to a single-cycle pulse, while the propagation of the imaginary pulse changes in the opposite direction, from single-cycle to 1½-cycle. In all cases, the characteristic parameter of helical pulses is $q_2 = 50q_1$. The propagation characteristics of the TM pulse are the same as in the TE case

presented here, except that the electric (magnetic) field is replaced by a magnetic (electric) field.

As the $l=0$ TE helical pulses (FD) propagates, the variation of its real and imaginary pulse is shown in Figure S1. It can be seen that the imaginary pulse has three wave flaps in the electric field E_θ at $t=0$, and with the propagation of the pulse, at $t=30q_1/c$, the first flap disappears and the third gradually increases. The real pulse propagates in free space with similar properties of wave flap transformation. At $t=0$, there are two electric field E_θ flaps, as the pulse continues to propagate, the first flap disappears and the third gradually increases. From the above analysis, it can be seen that the helical pulses in the process of propagation in free space, there is the phenomenon of wave flap transformation between two and three flaps. And in the time domain waveform it is immediately clear that as the pulses propagate beyond the q_2 parameter, the real pulse evolves from single-cycle to 1½-cycle, while the imaginary pulse evolves from 1½-cycle to single-cycle.

As the $l=1$ TE helical pulses propagates, the variation of its real and imaginary pulse is shown in Figure S2. The real part of the pulse is consistent with the imaginary part of the pulse at $l=0$, which changes from two flaps to three flaps and is reflected in the time domain waveform as a change from single-cycle to 1½-cycle. The imaginary part of the pulse is consistent with the real part of the $l=0$ pulse, which changes from three flaps to two flaps, and the time domain waveform is reflected as a transition from 1½-cycle to single-cycle.

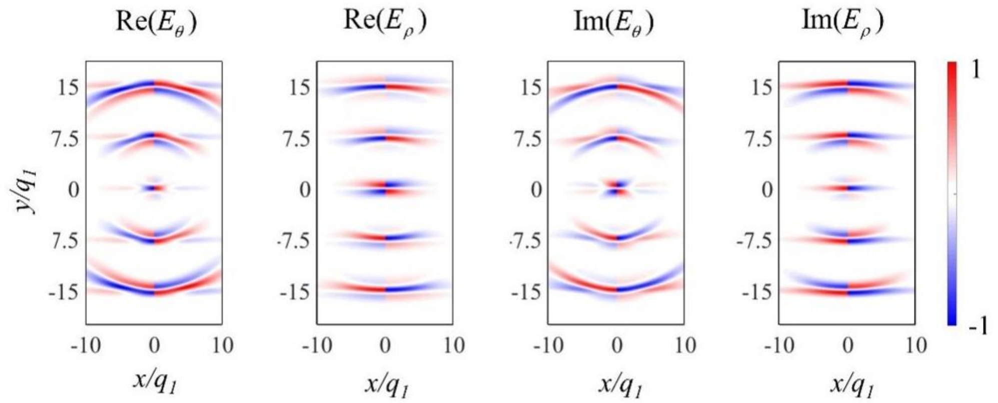

Figure S2 Time domain waveforms and the normalized longitudinal magnetic field components on the *y-z* cross-section of the real and imaginary parts of the TE $l=1$ helical pulses. Similarly, they exhibit a transition between single-cycle and 1½-cycle as they propagate *z*-direction. But in contrast to the case of conventional FD, the real pulse corresponds to a single-cycle pulse, transforming into a 1½-cycle pulse; while the imaginary pulse propagation changes in the opposite direction, from 1½-cycle to single-cycle.

We found that as l continues to increase (without considering the limitation of limited energy), the number of flaps of the pulse increases, the duration becomes shorter, and the overall frequency shifts. And as l increases, the field exhibits a more complex evolution, with the pulses being reshaped several times during propagation and the alternation of the positive and negative directions of its field shifting many times.

Finally, we can conclude that when $l=0$, its real and imaginary pulses of the electric field are embodied as 1½-cycle and single-cycle, respectively, while during the propagation process, 1½-

cycle imaginary pulse will transform into single-cycle, while the real pulse will transform from single-cycle to 1½-cycle. Single-cycle and 1½-cycle in the time domain waveform correspond to two and three flaps in the cross-section diagram of the field, respectively. And when $l = 1$, the case of the real and imaginary part of the pulse is exactly the opposite of the even case, where single-cycle (1½-cycle) corresponding to the real part becomes 1½-cycle (single-cycle) during propagation, while 1½-cycle (single-cycle) corresponding to the imaginary part propagating into single-cycle (1½-cycle), with the wave flaps corresponding to the time domain waveform, respectively.

Supplementary Note 3:

Intensity and phase distribution of TE helical pulses.

This note presents a step-by-step derivation of an analytical expression for the time-frequency Fourier transform of the helical pulses. The following Fourier transform pair is used:

$$\mathbf{F}(\mathbf{r},\omega)=\int_{-\infty}^{\infty}e^{i\omega t}\mathbf{F}(\mathbf{r},t)dt \quad \mathbf{F}(\mathbf{r},t)=\frac{1}{2\pi}\int_{-\infty}^{\infty}e^{-i\omega t}\mathbf{F}(\mathbf{r},\omega)d\omega \tag{S25}$$

The transverse electric (TE) fields are given by the Eq.(S9) and Eq.(S10), we first work with the electric field component E_θ. From now on and for clarity we will refer to the electric field as E, but we actually mean that we are using the E_θ component. The real and imaginary parts of the field are quite complex expressions to compute the Fourier integral. Thus, and because of the linearity of the integral operator, we will calculate the Fourier transform of the complex field and then we will take the real and imaginary parts from the equations

$$E_{\mathrm{re}}(\omega)=\frac{E(\omega)+E^*(-\omega)}{2} \quad E_{\mathrm{im}}(\omega)=\frac{E(\omega)-E^*(-\omega)}{2i} \tag{S26}$$

Returning now to Eq.(S10), it is apparent that we can apply Jordan's lemma[5] since the power of t on the denominator is much bigger than that of the numerator. Thus, the Fourier transform is given by the integral residues on the upper and lower half-complex-plane. One only has to find the poles and determine when they are located in upper half or lower half-plane. It is apparent that $l=0$ case has two triple poles, while $l=1$ case has one more double pole than $l=0$. We start by writing the poles

$$\begin{aligned} t_1 &= -\left(\sqrt{-(q_1-q_2-2\rho+2iz)(q_1-q_2+2\rho+2iz)}+i(q_2+q_1)\right)\Big/2c \\ t_2 &= -\left(-\sqrt{-(q_1-q_2-2\rho+2iz)(q_1-q_2+2\rho+2iz)}+i(q_2+q_1)\right)\Big/2c \\ t_3 &= -(iq_1-z)/c \end{aligned} \tag{S27}$$

It has been proven that all the poles are located in the lower half complex plane, so the integral can be calculated from the integral residues

$$\int_{-\infty}^{+\infty}e^{i\omega t}E(t)dt=2\pi i\sum_i Res\left[e^{i\omega t}E(t),t_i\right]I \tag{S28}$$

With I denoting the sign of the contour (positive for anticlockwise). In general, we consider the following three cases

For $\omega>0$

$$\begin{cases} E(\omega)=0 \\ E^*(-\omega)=\left(-2\pi i\sum_i Res\left[e^{-i\omega t}E(t),t_i\right]\right)^* \end{cases} \tag{S29}$$

For $\omega<0$

$$\begin{cases} E(\omega) = \left(-2\pi i \sum_i Res\left[e^{i\omega t} E(t), t_i \right] \right) \\ E^*(-\omega) = 0 \end{cases} \tag{S30}$$

For $\omega = 0$, the choice of the contour does not alter the result and knowing that there are no poles in the upper half-plane and the line of real values, the calculus of residues immediately gives the answer of having a zero integral. That is, there are no DC components in the field. However, above three cases can be simplified to a single line, given that for the Fourier transform of a real function, it holds that $F(\omega) = F^*(-\omega)$. For convenience, we can define the following expressions

$$\varepsilon = (q_1 - q_2 + 2iz) \quad A = -(\varepsilon - 2\rho)(\varepsilon + 2\rho) \tag{S31}$$

There are no poles in the upper half-plane, but reversal of the sign of ω is equivalent to integrating over the path of the lower half-plane. Finally, the residues are

$$l = 0 \begin{cases} Res\left[e^{i\omega t} E(t), t_1 \right] = \dfrac{2e^{\frac{\omega}{2c}\left(q_1 + q_2 - i\sqrt{A}\right)} \omega\rho(2ci - \omega\sqrt{A})}{c^3 A^{3/2}} \\ Res\left[e^{i\omega t} E(t), t_2 \right] = -\dfrac{2e^{\frac{\omega}{2c}\left(q_1 + q_2 + i\sqrt{A}\right)} \omega\rho(2ci + \omega\sqrt{A})}{c^3 A^{3/2}} \end{cases}$$

$$l = 1 \left\{ Res\left[e^{i\omega t} E(t), t_3 \right] = -e^{i\theta} \dfrac{e^{\frac{\omega}{c}(q_1 + iz)} (2\omega\rho^4 - \omega\rho^2 + 4c\varepsilon)}{c^2 \rho^4} \right. \tag{S32}$$

The Fourier transform for the TE pulse can be given by the Eq.(S28). It is clear that the magnetic fields satisfy the necessary conditions to apply Jordan's lemma and have the same poles as the electric field. Hence, the exact same approach can be used, leading to the frequency domain expressions for the magnetic field. The TM pulses can be obtained by exchanging the electric and magnetic fields of the TE mode by Eq.(S20). Since in the time domain, the real and imaginary parts form Hilbert transform pairs, which means that they also share the same spectrum in the frequency domain, varying only in phase, as can be seen from the following relation

$$\mathbf{E}_{\text{im}}(\omega) = i\,\text{sgn}(\omega)\mathbf{E}_{\text{re}}(\omega) \quad \mathbf{H}_{\text{im}}(\omega) = i\,\text{sgn}(\omega)\mathbf{H}_{\text{re}}(\omega)$$
$$\text{sgn}(\omega) = \begin{cases} 1, & \omega > 0 \\ -1, & \omega < 0 \\ 0, & \omega = 0 \end{cases} \tag{S33}$$

Above we have analytically obtained how to derive the frequency domain expressions for the real and imaginary parts of each field component of the TE and TM pulses, and based on the expressions, we can analyze the spectral phase of the pulses. Constrained conditions based on limited energy, we take two different sets of TE helical pulses with $l = 0$ and 1 to study and analyze the intensity and phase distribution characteristics of each field component based on the frequency domain expressions calculated by MATLAB tools to more intuitively study and analyze the phase variation law of helical pulses.

The $l=0$ TE helical pulses has only one component: the transverse electric field E_θ and we derive the phase expressions for this field components

$$E_\theta^{\text{re}}(\omega) = -2i\pi\omega\rho e^{-\frac{\omega(q_2+q_1)}{2c}} \frac{e^{\frac{i\omega}{2c}\sqrt{A}}(2ic+\omega\sqrt{A}) - e^{-\frac{i\omega}{2c}\sqrt{A}}(2ic-\omega\sqrt{A})}{c^2 A^{3/2}} \tag{S34}$$

Then we continue to discuss the $l=1$ TE helical pulses. At this time, there are two electrical components, which are transverse electric field E_ρ, E_θ. We derive the phase expressions for each of these two field components

$$E_\rho^{\text{re}}(\omega) = \frac{\pi\omega}{c^2} e^{i\theta} \left[\frac{2i}{\sqrt{A}} e^{\frac{\omega(q_2+q_1)}{2c}} \left(\frac{e^{\frac{i\omega}{2c}\sqrt{A}}}{(i\sqrt{A}+\varepsilon)} + \frac{e^{-\frac{i\omega}{2c}\sqrt{A}}}{(i\sqrt{A}-\varepsilon)} \right) - \frac{e^{\frac{\omega}{c}(q_1+iz)}}{\rho^2} \right] \tag{S35}$$

$$\begin{gathered} E_\theta^{\text{re}}(\omega) = \frac{-\pi\omega i}{c^2} e^{i\theta} \left[\frac{2i}{cA} e^{\frac{\omega(q_2+q_1)}{2c}} \left(\frac{e^{-\frac{i\omega}{2c}\sqrt{A}}\alpha_1}{\beta_1} + \frac{e^{\frac{i\omega}{2c}\sqrt{A}}\alpha_2}{\beta_2} \right) - \frac{e^{\frac{\omega}{c}(q_1+iz)}}{\rho^2} \right] \\ \alpha_1 = 8\omega\rho^4 + icA^{3/2} - c\varepsilon^3 + 2\rho^2\left(-4ic\sqrt{A} - i\omega\sqrt{A}\varepsilon + \omega\varepsilon^2\right) \\ \alpha_2 = 8\omega\rho^4 - icA^{3/2} - c\varepsilon^3 + 2\rho^2\left(4ic\sqrt{A} + i\omega\sqrt{A}\varepsilon + \omega\varepsilon^2\right) \\ \beta_1 = -\varepsilon^3 + 4\rho^2\varepsilon + i\sqrt{A}\varepsilon^2 \quad \beta_2 = -\varepsilon^3 + 4\rho^2\varepsilon - i\sqrt{A}\varepsilon^2 \end{gathered} \tag{S36}$$

Taking the helical pulses with $q_2 = 10q_1$ as an example, according to the above formula, we draw the intensity of the $z=0$ plane in Figure S3. As a result of their short cycle nature, helical pulses are considered to be ultrabroad bandwidth pulses, a number of intriguing properties of the helical pulses can be inferred from the Fourier decomposition.

We show the spectral distribution of the transverse electric field at different radii in the $z=0$ plane with the normalized two-dimensional spectral distribution of the transverse electric field. From the radial distribution of spectral power of the pulse at focus, it can be seen that the space-frequency coupling becomes apparent, high frequency components dominate the center and lower frequency components become prevalent as ρ increases, which leads to a separation of the wavelengths with the maxima of the longer wavelengths located at larger radii and the shorter wavelengths closer to the center at any propagation distance. From the two-dimensional spectrum figure, when $l=1$, the maximum value of the transverse electric field at all frequency points is located at $\rho=0$. With the increase of ρ, the spectrum of E_θ decreases and then increases, forming a band with zero value in the spectrum. This is because the position of $\rho=0$ is the convergence point of two vortices, at which the field value reaches its maximum, and the properties of the double helix structure make E_θ and E_θ uniformly exist at that point. When l is zero, due to its rotational symmetry property, so is zero at $\rho=0$. As ρ increases, the value of each frequency point first increases and then decreases. Comparing the spectra at different ρ can also be verified: the larger the ρ position, the spectrum is mainly concentrated in the low frequency region, i.e., as ρ increases, the spectral maxima move to the low frequency.

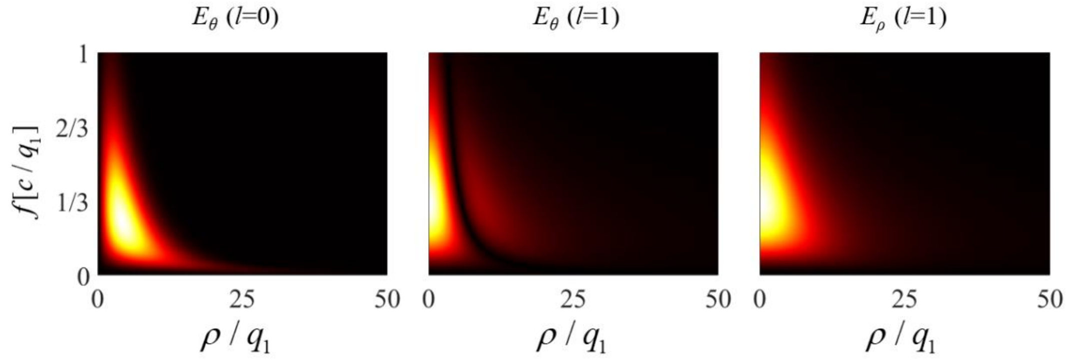

Figure S3 Fourier spectrum of the real TE helical pulses with different orders. The figure shows the normalized two-dimensional spectral distribution of the electric field at $z=0$ plane.

Under the same parameter settings, we draw the intensity and phase distribution at $f=\frac{1}{6}c/q_1, \frac{1}{3}c/q_1, \frac{1}{2}c/q_1$ on the $z=-20q_1$, 0 and $10q_1$ cross-section, respectively. Figure S4 shows the E_θ component of $l=0$ TE helical pulses, while Figures S5 and S6 respectively display the E_ρ and E_θ components of $l=1$ TE helical pulses.

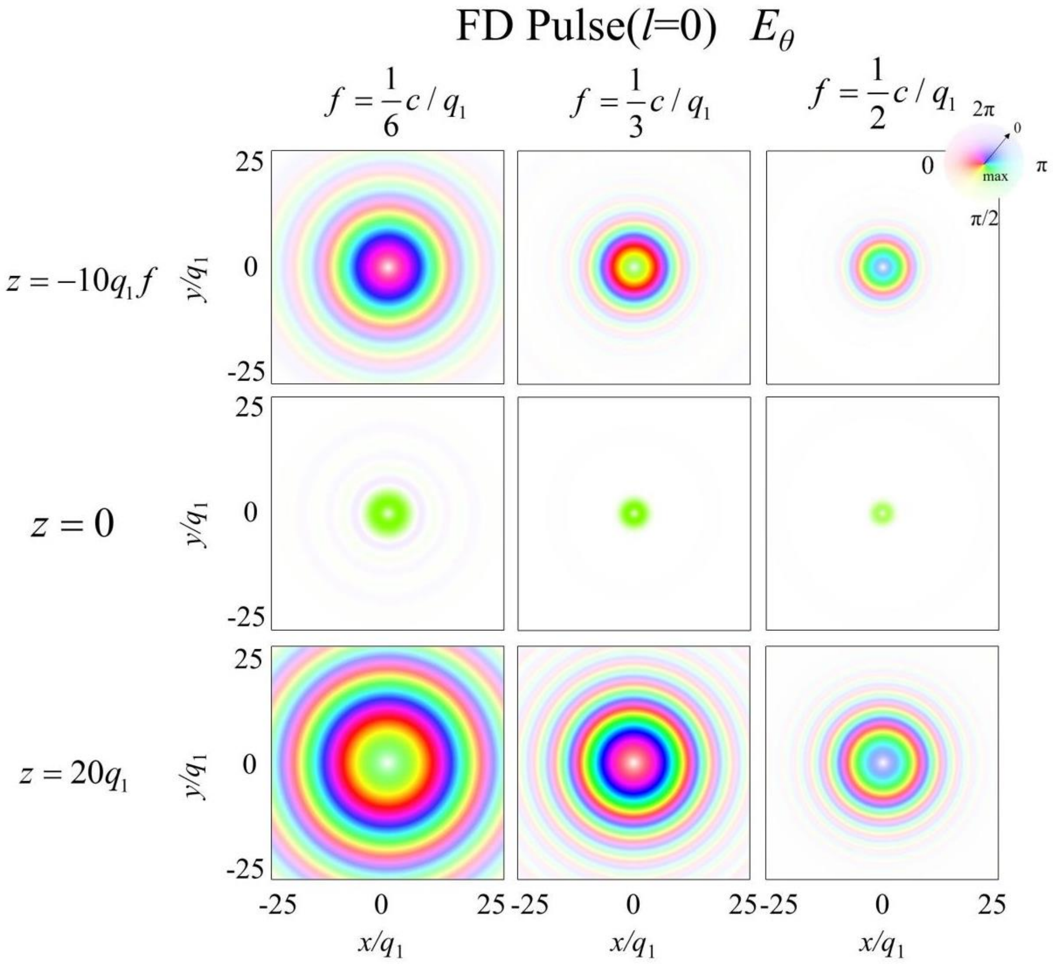

Figure S4 Intensity and phase distribution of TE FD Pulses. With the Rayleigh range $q_2=10q_1$ on

$z=-10q_1$, $z=0$ and $z=20q_1$ at frequency $f=\frac{1}{6}c/q_1$, $f=\frac{1}{3}c/q_1$ and $f=\frac{1}{2}c/q_1$.

The $l=0$ TE helical pulses has one electrical components, it shows a bright ring distribution around the central dark nucleus, while the phase corresponding to the central dark nucleus with intensity 0 shows a phase singularity in the center, and excluding the phase singularity, the phase distribution in the whole plane no longer changes. We can also see that the vortex phase factor does not come into play when $l=0$, so there is no vortex phase in this case. In the central region (energy concentration area) on the $z=0$ plane, different frequencies exhibit the same phase, indicating that they superpose in phase on this plane. Furthermore, at $z=-10q_1$ and $z=20q_1$, points with identical phase connect to form mutually nested concentric rings, providing strong evidence for the existence of Gouy phase shift in the FD pulse. The state change of phase also reflects, from another perspective, the significant variations in the waveform during transmission. The mutual transformation between the $1\frac{1}{2}$-cycle and single-cycle of the pulse is precisely based on this phenomenon.

From the Figure S4 and Figure S5, we can see that the center points of all components are phase singularities with abrupt phase changes, and each cycle has a 2π change in phase on each plane. The phase distribution of the E_θ component is consistent with the E_ρ components, but the intensity distribution is slightly different, as the intensity distribution of E_θ component shows several bright ring distributions around the central dark core. Moreover, as the distance from the $z=0$ plane increases, the bright rings of the amplitude distribution of each frequency become more prominent. The phase distribution of E_θ has a phase mutation in the circle centered on the center in addition to the phase singularity in the plane, and the 2π phase change is realized in the opposite rotation direction inside and outside the mutation circle, which leads to two places where the intensity distribution is zero, as a bright circle with the center singularity dug out and a bright ring in the outer circle. Similar to the case of $l=0$, on the $z=0$ plane, the different frequencies of the two components exhibit the same phase or phase variation at the same azimuthal angle, indicating the existence of in-phase conditions within the helical pulses pulse as well.

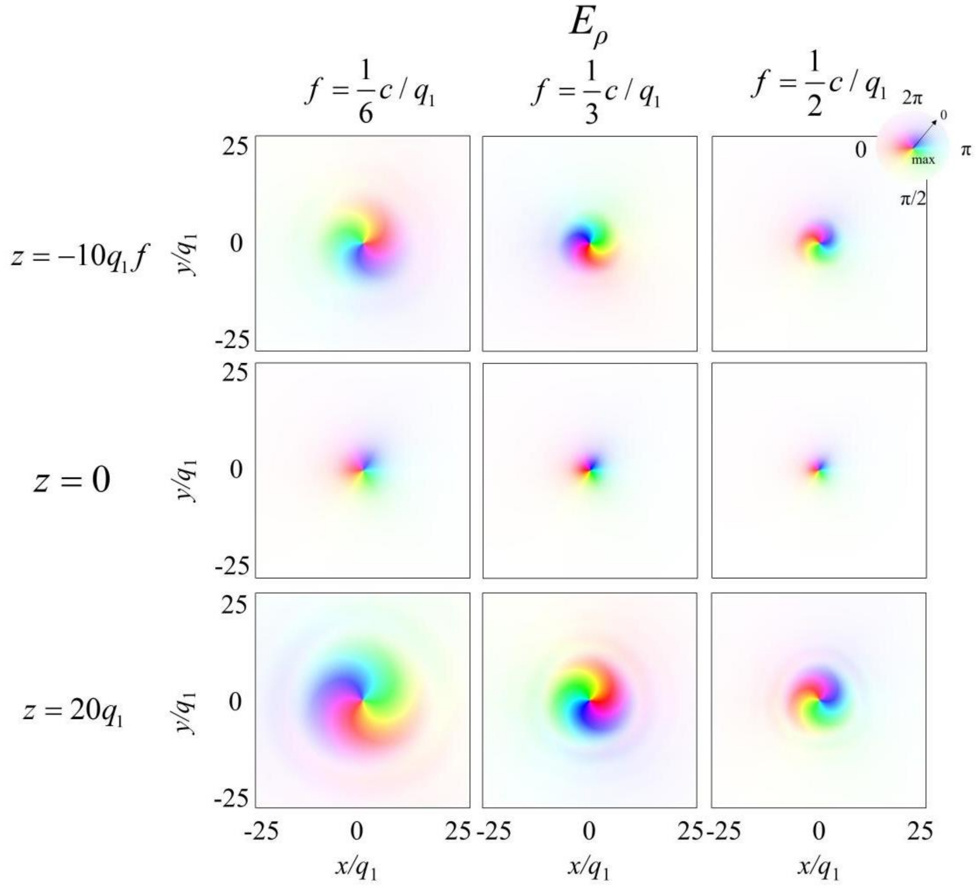

Figure S5 Intensity and phase distribution of component E_ρ of $l=1$ TE helical pulses. With the Rayleigh range $q_2=10q_1$ on $z=-10q_1$, $z=0$ and $z=20q_1$ at frequency $f=\frac{1}{6}c/q_1$, $f=\frac{1}{3}c/q_1$ and $f=\frac{1}{2}c/q_1$.

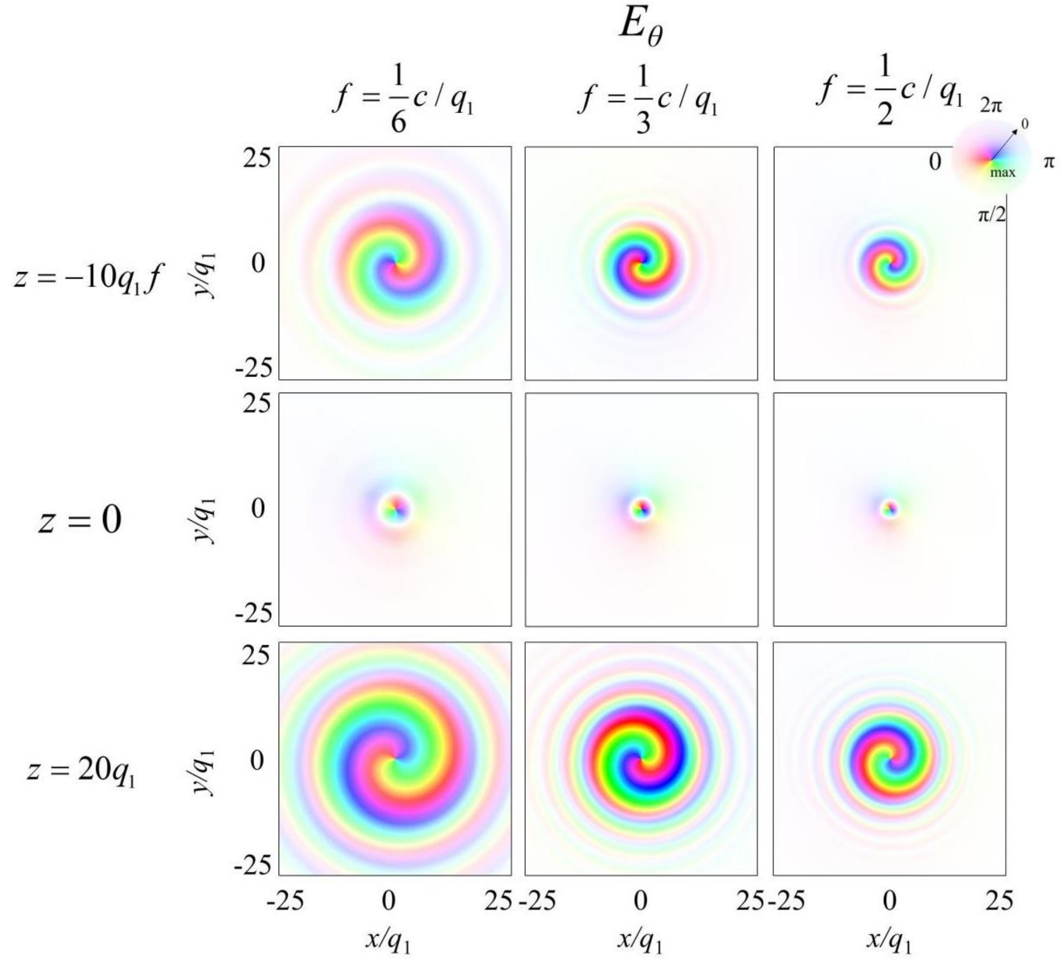

Figure S6 Intensity and phase distribution of component E_θ of $l=1$ TE helical pulses. With the Rayleigh range $q_2=10q_1$ on $z=-10q_1$, $z=0$ and $z=20q_1$ at frequency $f=\frac{1}{6}c/q_1$, $f=\frac{1}{3}c/q_1$ and $f=\frac{1}{2}c/q_1$.

Supplementary Note 4:
Calculation method of vortex and antivortex of TE helical pulses.

This note presents the calculation method of vortex and antivortex. Since our singularity is defined as the zero-point position of the field value, the search for the singularity position is transformed into solving a system of equations

$$\begin{cases} E_\rho(\rho,\theta,z,t)=0 \\ E_\theta(\rho,\theta,z,t)=0 \end{cases} \tag{S37}$$

Drawing inspiration from the analysis of phase trajectories near singularity in nonlinear autonomous systems of ordinary differential Eq.[6], we can apply streamline analysis to the electric field in the vicinity of singularities. By integrating the criteria for streamline classification in the proximity of singularities in phase planes, we can categorize the electric field streamlines in the region surrounding the singularities.

First, the electric field equations are treated as a system of ordinary differential equations.

$$\frac{\mathrm{d}x}{\mathrm{d}\zeta}=E_x(x,y) \tag{S38}$$

$$\frac{\mathrm{d}y}{\mathrm{d}\zeta}=E_y(x,y) \tag{S39}$$

In the context of the nonlinear differential equation system, E_x and E_y represent the expressions for the two transverse components of the electric field, while $x(\zeta)$ and $y(\zeta)$ are parametric equations with ζ as the parameter and $\zeta \in (-\infty,\infty)$. In this context, ζ solely corresponds to the differential equation system and is not relevant to our study.

According to the definition of a nonlinear differential equation system, singularities occur at solutions that simultaneously satisfy $\mathrm{d}x/\mathrm{d}\zeta=0$ and $\mathrm{d}y/\mathrm{d}\zeta=0$, which aligns with our research scenario. In the study of singularities in nonlinear autonomous systems of ordinary differential equations, several classical singularity types exist, including star node, two-directional node, one-directional node, saddle point, focus, and center. Among these, centers and saddle points are two significant singularity configurations. In magnetization dynamics, centers and saddle points are referred to as vortices and antivortices. For the sake of uniformity, in the remaining part of this discussion, we will uniformly use the terms "vortex" and "antivortex" to refer to them. Due to limitations in space and the scope of the study, only vortices and antivortices will be discussed here; further details can be found in reference[6]. The singularity structures of vortices and antivortices are depicted in Figure S7(a) and (b), respectively.

The topological protection of vortices and antivortices is primarily manifested in their resistance to uniform perturbations. Suppose an infinitesimally small, uniform perturbation is introduced into a field containing vortices or antivortices; such a perturbation cannot destroy the singularities because it is always possible to find an opposite field value nearby to cancel out the

perturbation, thereby moving the singularities to new locations. The only feasible way to eliminate singularities through uniform perturbations is to increase the perturbation intensity, thereby inducing the merging and annihilation of vortices and antivortices or vortices of opposite chirality. In our research, the center of the wave packet consistently maintains a topological structure of double-helical vortices. This topological characteristic of the wave field ensures the robustness of singularities in the face of small field perturbations, which is crucial for the effective propagation of information.

Before examining the relationship between the singularity structures and the system of differential equations, it is necessary to assume a location for the singularity. Here, we set the singularity at the point (0,0). Other scenarios can be discussed by translating the singularity to the origin through coordinate transformations, this is allowed. Next, we need to perform a first-order Taylor expansion of the system of differential equations at the point (0,0). The expansion expressions are given in Eq.(S40) and Eq.(S41).

$$\frac{\mathrm{d}x}{\mathrm{d}\zeta} = E_x(x,y) = ax + by + \varphi(x,y) \tag{S40}$$

$$\frac{\mathrm{d}y}{\mathrm{d}\zeta} = E_y(x,y) = cx + dy + \psi(x,y) \tag{S41}$$

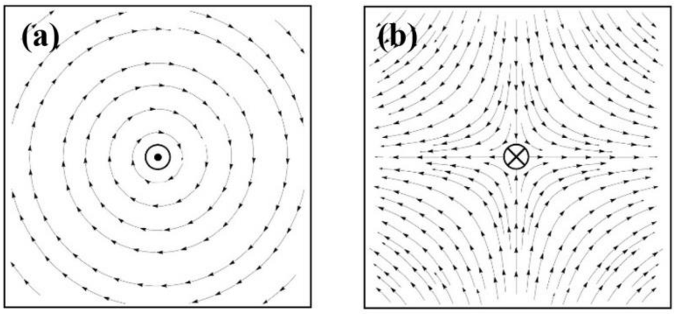

Figure S7 Schematics of a magnetic (a) vortex and (b) an antivortex structure. In the core of the (anti)vortex, the magnetization is perpendicular to the plane.

In the given context, φ and ψ represent higher-order terms for x, y, while a, b, c, and d denote the Taylor expansion coefficients. For the determination of an antivortex, the criteria are relatively straightforward and require the simultaneous satisfaction of the following three conditions,

(1) The coefficient matrix constructed from the Taylor expansion coefficients

$$\begin{pmatrix} a & b \\ c & d \end{pmatrix} \tag{S42}$$

has only real eigenvalues that are opposite in sign to each other.

(2) The function $\varphi(x,y)$ and $\psi(x,y)$ are differentiable with respect to both x and y within a certain neighborhood of the point (0,0).

(3) $\varphi(x,y)$, $\psi(x,y)=o(r), r=\sqrt{x^2+y^2}\to 0$

For vortices, the determination criteria are more stringent. Two classical approaches are the successor function method and the formal series method, both established by Poincaré[7]. Both methods present computational complexities that have remained a focus of attention for many scholars, leading to the development of various methods such as those integral factor methods[8] and invariant curve method[9], etc. Here, we use a determination method[6], which combines mechanical principles and geometric properties. The specific contents of this method include the following two conditions,

(1) The coefficient matrix constructed from the Taylor expansion coefficients

$$\begin{pmatrix} a & b \\ c & d \end{pmatrix} \tag{S43}$$

has a pair of conjugate complex eigenvalues.

(2) Let $\Gamma=\{\mathrm{F}^2+\mathrm{G}^2=g\}$, where $\mathrm{F}=ax+by+\varphi(x,y)$ and $\mathrm{G}=cx+dy+\psi(x,y)$, and it holds that

$$\kappa=\oint_{\Gamma}\frac{\mathrm{d}x}{\mathrm{d}\zeta}\frac{\mathrm{d}^2x}{\mathrm{d}\zeta^2}+\frac{\mathrm{d}y}{\mathrm{d}\zeta}\frac{\mathrm{d}^2y}{\mathrm{d}\zeta^2}\mathrm{d}s=0 \tag{S44}$$

for all sufficiently small values of g .

The determination of singularities based on differential equation systems is also involved in areas such as fluid mechanics and polarization singularity theory in optics[10].

We selected multiple different singularities at different times for numerical calculations and determined their types, the judgment results were consistent with our expected results, indicating that the singularities present in helical pulses are vortices and antivortices.

The dimensional characteristics of the double helix structure are primarily composed of two parameters: pitch and radius. Among them, the size of the radius is jointly influenced by both parameters q_1 and q_2 , while the pitch is solely affected by q_1 . This phenomenon can be explained by the fact that q_1 primarily has a significant impact on the operating frequency band, manifesting as the duration and spatial extent of the pulse in the time-domain waveform. In contrast, q_2 does not directly influence the operating frequency band, hence the variation in pitch is solely dependent on q_1. The variation laws of pitch and radius with respect to changes in q_1 and q_2 can be visually represented through Figure S8.

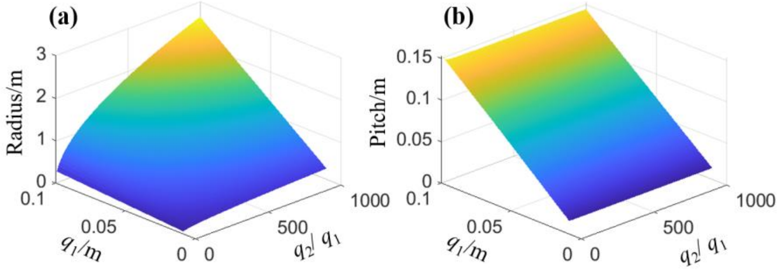

Figure S8 The variation laws of pitch and radius with respect to changes in q_1 and q_2. With the increasing values of parameters q_1 and q_2, the radius exhibits a trend of enlargement. In contrast, the pitch is solely influenced by parameter q_1, demonstrating no significant correlation with parameter q_2.

We constructed a formula for calculating the winding number of double helical singularities based on the existing mathematical formula for winding definition, as shown in Eq.(S45). In this formula, the symbol α represents the angle between the line connecting the two singularities and the x-axis, the schematic illustration is provided in Figure S9.

$$W = \frac{1}{2\pi}\int_{-\infty}^{-\infty} \frac{\partial \alpha(t,z)}{\partial z} dz \tag{S45}$$

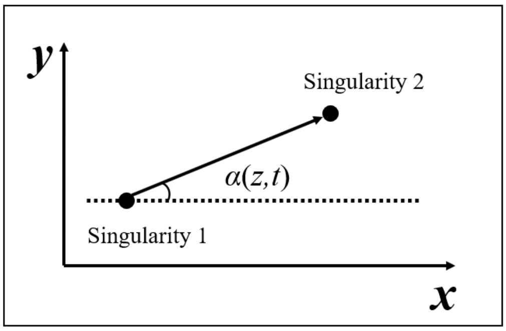

Figure S9 Illustration of the computation for Eq.(S45). Singularity 1 and Singularity 2 correspond respectively to the left-handed vortex singularity point and the right-handed vortex singularity point in the vicinity of the wave packet center. The symbol α represents the angle between the line connecting the two singularities and the x-axis.

We analyzed the dynamic evolution of the winding number over time and represented it in Figure S10. The computational results approaching 1 indicate that the two singularity lines have completed one full winding cycle.

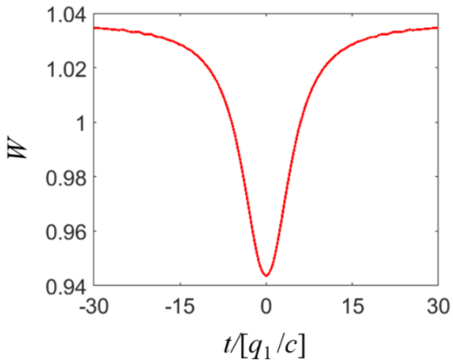

Figure S10 The law of winding number variation over time, with the Rayleigh range $q_2 = 10q_1$.

When $l \geq 2$, singularities solely exist at the propagation axis $\rho = 0$ and manifest as antivortex, with the number of lobes in the antivortex precisely equal to $2l$. Taking Figure S7(b) as an example, Figure S7(b) is identical to the singularity scenario when $l = 2$. We define the boundaries of the lobes based on vector arrows pointing towards and away from the singularities. In this case, Figure S7(b) exhibits four lobes. By analogy, as l increases, the number of lobes

synchronously grows at a rate of $2l$.

Supplementary Note 5:
Measures of space-time nonseparability of TE helical pulses.

The similarity between classical and quantum worlds allows us to apply quantum tools to describe many of the properties of classical nonseparable states of light, with an increasing number of intriguing higher-dimensional structured light modes (space-time nonseparable pulse) enabling the simulation of an increasing number of higher-dimensional quantum states, which allows the transfer of more quantum tools to classical fields. Over the past century, an extended toolbox has been developed to quantify the quality of a quantum entangled state and characterize the spatiotemporal propagation dynamics, including state tomography, density matrix, fidelity, concurrence, etc. The quantumlike measures introduced here also have a clear physical meaning related to the propagation dynamics of spatiotemporal pulses. In particular, the density matrix contains the full information of the correlation between the spectral and spatial states. The fidelity quantifies the similarity between two pulses. On the other hand, concurrence and EoF quantify the overlap of spectral states with spatial states.

Electromagnetic spatiotemporal pulses can be described by a bipartite state with the space and time DoFs, which can be expressed as a product of a spatial mode and a temporal (spectral) function, corresponding to a general high-dimensional bipartite state. However, "electromagnetic directed-energy pulse trains" (EDEPTs) as space-time nonseparable exact solutions of Maxwell's equations, whose family includes pulses such as FDs, are typical examples of STNS (space-time nonseparability) pulses, that cannot be expressed as products of spatial and temporal functions, corresponds to the maximally entangled nonseparable state. Since vortex beams carrying helical phase, as classical analog quantum states associated with orbital angular momentum (OAM), can also be represented as space-polarization nonseparable states, we believe that the new electromagnetic pulses proposed by introducing vortex phase factors in FD pulse still have space-time nonseparability, and we quantize STNS with measures of fidelity, coherence, and entanglement of formation in quantum measurements. We can introduce two sets of states to describe STNS in pulses: (1) Spectral states $\left|\lambda_i\right\rangle(i=1,2,\ldots,n)$ are states of light of defined wavelength λ_i and with defined radial position ($r_{\lambda i}$) of peak intensity; (2) spatial states $\left|\eta_i\right\rangle$ are states of light located at the position with a defined radial ratio of $\eta_i = r / r_{\max}$, where $r_{\max}$ is the radial position at which the total intensity of the light field reaches its maximum. Based on the prior theory, the introduction of spatial and spectral sets of states allows us to distinguish similar broadband waves. As an example, we consider six pulses with different STNS, a wideband LG (Laguerre-Gaussian) beam[11], a fundamental FD, a helical pulses ($l=1$), a STBG (spatiotemporal Bessel-Gaussian) beam[12], a STBV (spatiotemporal Bessel vortices)[13] and a STXV (ultrafast X vortices)[14].

As illustrated by the corresponding spatial and spectral states, all pulses exhibit very different spatial-spectral structure and propagation dynamics. For the FD pulse and the helical pulses, the spectral states are coincident with the corresponding spatial states upon propagation, as Figure S11(b1) and (c1) show. In contrast, the wideband LG beam, two spatiotemporal Bessel beams and

the ultrafast X vortices' corresponding spectral and spatial states are naturally separated (see Figure S11(a1) and (d1) - (f1)), and the spatial-spectral structure of these beams varies dramatically as they propagate. The difference among the two types of beams can be emphasized further in the $\eta - z$ plane. Here, as expected, the spectral states of the FD pulse and the helical pulses (Figure S11(b2) and (c2)) are z invariant and coincident with the corresponding spatial states. The crucial characteristic induced by the space-time nonseparable pulse is the isodiffraction nature, whereby the spatial distribution of different spectral components in the transverse plane does not suffer distortion upon propagation. On the other hand, the profile of the remaining four beams (Figure S11(a2) and (d2)-(f2)) suffer substantial distortion as illustrated by the trajectories of the spectral states. This is a direct result of the noncoincidence of spectral and spatial states.

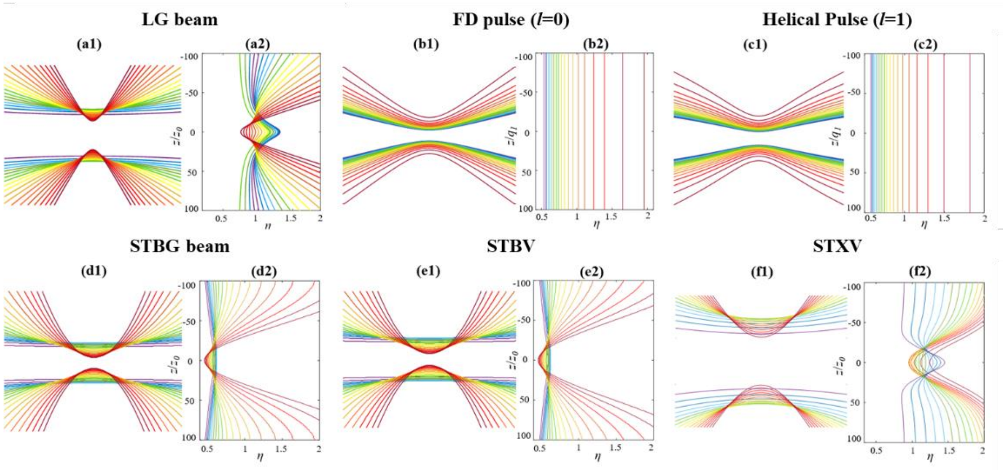

Figure S11 The propagation profiles of spectral ($|\lambda_i\rangle$), spatial ($|r_i\rangle$) states and the $\boldsymbol{\eta}$ - $\boldsymbol{z}$ map of different pulses: the wideband LG beam (a1-a2), the FD pulse (b1-b2), the $l=1$ helical pulses (c1-c2), the spatiotemporal Bessel-Gaussian beam (d1-d2), the spatiotemporal bessel vortices (e1-e2) and the ultrafast X vortices (f1-f2). In this illustration, we have considered 20 different spectral and spatial states, $|\lambda_i\rangle$ and $|\eta_i\rangle$ ($i = 1, 2, ..., 20$), where radial ratios are selected as $\eta_i = r_{\lambda_i} / r_{\max}$.

We summarize the results of fidelity, concurrence and entanglement measures in Table S1. The corresponding results for the ideal FD and helical pulses show that both pulses exhibit near-maximum "entanglement", and the high EoF for the FD and helical pulses reveals that they both exhibit a strong STNS. The root cause of the nonseparability discussed here lies in the fact that the time variable and the spatial variable in the formula cannot be written as the product of a time function and a spatial function through the method of separation of variables. Eq.(S7) demonstrates this point quite notably. Consequently, the ideal FD and helical pulses derived from Eq.(S7) exhibit nearly maximal entanglement.

Table S1 shows a parameter comparison of various kinds of pulses. N-Fid., N-Conc., and N. EoF, fidelity, concurrence, and EoF in intensity-normalized measurement, respectively.

**Pulse**	**W.LG**	**FD**	**helical pulses**	**STBG**	**STBV**	**STXV**
**N-Fid.**	0.0016	1	1	0.0011	0.0025	0.0028
**N-Conc.**	0.7079	0.9975	0.9977	0.7065	0.6973	0.7050
**N-EoF**	0.2233	0.9807	0.9857	0.2227	0.2185	0.2220

Supplementary Video

Supplementary Movies 1: Electric field vector evolution of helical pulses. Dynamic evolution of the electric field projected on the different cross section upon helical pulses propagating. The animation demonstrates the electric field vector images at different moments from $t = -3q_1$ to $t = 3q_1$ on the $z = 0$ plane, with the parameter settings of $q_1 = 0.01$ and $q_2 = 10q_1$. The direction of the electric field vector arrows is correlated with the azimuth φ angle, and the specific color legend can be referred to in Figure 2 of the main text.

Supplementary Movies 2: Electric field vector and singularity line evolution of helical pulses. The animation showcases the dynamic evolution of electric field vectors and singularity lines across different cross-sections of the electric field. The parameters are set as $q_1 = 0.01$ and $q_2 = 10q_1$, and the time range varies from $t = -15q_1$ to $t = 15q_1$. We use red to mark the vortex core of the right-handed vortex field and blue to mark the vortex core of the left-handed vortex field, The displayed cross-sections are at $z = -100q_1$, $-50q_1$, 0, $50q_1$ and $100q_1$. The direction of the electric field vector arrows is correlated with the azimuth φ angle, and the specific color legend can be referred to in Figure 2 of the main text.

Supplementary Movies 3: Spatiotemporal dynamic evolution of the three-dimensional structure and singularity upon helical pulses propagating. The animation demonstrates the dynamic evolution of the three-dimensional topological structure of the E_ρ component along with the singularity lines. The parameters are set as $q_1 = 0.01$ and $q_2 = 10q_1$, with the time range varying from $t = -15q_1$ to $t = 15q_1$. In the three-dimensional topological structure, the red and blue semi-transparent surfaces represent the normalized isosurfaces of the E_ρ component where $E_\rho = \pm 0.01$, respectively. We use red to mark the vortex core of the right-handed vortex field and blue to mark the vortex core of the left-handed vortex field.

[1] R. W. Ziolkowski, "Localized transmission of electromagnetic energy," *Physical Review A,* vol. 39, no. 4, p. 2005, 1989.

[2] P. Hillion, "Generalized phases and nondispersive waves," *Acta Applicandae Mathematica,* vol. 30, pp. 35-45, 1993.

[3] J. Lekner, "Localized electromagnetic pulses with azimuthal dependence," *Journal of Optics A: Pure and Applied Optics,* vol. 6, no. 7, p. 711, 2004.

[4] R. Hellwarth and P. Nouchi, "Focused one-cycle electromagnetic pulses," *Physical Review E,*

vol. 54, no. 1, p. 889, 1996.

[5] G. B. Arfken, H. J. Weber, and F. E. Harris, *Mathematical methods for physicists: a comprehensive guide*. Academic press, 2011.

[6] W. E. Boyce, R. C. DiPrima, and D. B. Meade, *Elementary differential equations and boundary value problems*. John Wiley & Sons, 2021.

[7] H. Poincaré, "Mémoire sur les courbes définies par une équation différentielle," *Journal de mathématiques pures et appliquées,* vol. 7, pp. 375-422, 1881.

[8] A. Algaba, M. Díaz, C. García, and J. Giné, "Center problem for generic degenerate vector fields," (in English), *Nonlinear Anal.-Theory Methods Appl.,* Article vol. 214, p. 23, Jan 2022, Art no. 112597, doi: 10.1016/j.na.2021.112597.

[9] L. G. S. Duarte and L. Da Mota, "An efficient method for computing Liouvillian first integrals of planar polynomial vector fields," (in English), *J. Differ. Equ.,* Article vol. 300, pp. 356-385, Nov 2021, doi: 10.1016/j.jde.2021.07.045.

[10] A. J. Vernon, M. R. Dennis, and F. J. Rodríguez-Fortuño, "3D zeros in electromagnetic fields," (in English), *Optica,* Article vol. 10, no. 9, pp. 1231-1240, Sep 2023, doi: 10.1364/optica.487333.

[11] Y. Shen, A. Zdagkas, N. Papasimakis, and N. I. Zheludev, "Measures of space-time nonseparability of electromagnetic pulses," *Physical Review Research,* vol. 3, no. 1, p. 013236, 03/12/ 2021, doi: 10.1103/PhysRevResearch.3.013236.

[12] M. Dallaire, N. McCarthy, and M. Piché, "Spatiotemporal bessel beams: theory and experiments," *Optics Express,* vol. 17, no. 20, pp. 18148-18164, 2009.

[13] A. Chong, C. Wan, J. Chen, and Q. Zhan, "Generation of spatiotemporal optical vortices with controllable transverse orbital angular momentum," *Nature Photonics,* vol. 14, no. 6, pp. 350-354, 2020.

[14] M. A. Porras and C. Conti, "Couplings between the temporal and orbital angular momentum degrees of freedom in ultrafast optical vortices," *Physical review A,* vol. 101, no. 6, p. 063803, 2020.
